# Psychological Processes Mediate the Impact of Familial Risk, Social Circumstances and Life Events on Mental Health

**DOI:** 10.1371/journal.pone.0076564

**Published:** 2013-10-16

**Authors:** Peter Kinderman, Matthias Schwannauer, Eleanor Pontin, Sara Tai

**Affiliations:** 1 Institute of Psychology, Health and Society, University of Liverpool, Liverpool, United Kingdom; 2 School of Health in Social Science, University of Edinburgh, Edinburgh, Scotland, United Kingdom; 3 School of Psychological Science, University of Manchester, Manchester, United Kingdom; Federal University of Rio de Janeiro, Brazil

## Abstract

**Background:**

Despite widespread acceptance of the ‘biopsychosocial model’, the aetiology of mental health problems has provoked debate amongst researchers and practitioners for decades. The role of psychological factors in the development of mental health problems remains particularly contentious, and to date there has not been a large enough dataset to conduct the necessary multivariate analysis of whether psychological factors influence, or are influenced by, mental health. This study reports on the first empirical, multivariate, test of the relationships between the key elements of the biospychosocial model of mental ill-health.

**Methods and Findings:**

Participants were 32,827 (age 18–85 years) self-selected respondents from the general population who completed an open-access online battery of questionnaires hosted by the BBC. An initial confirmatory factor analysis was performed to assess the adequacy of the proposed factor structure and the relationships between latent and measured variables. The predictive path model was then tested whereby the latent variables of psychological processes were positioned as mediating between the causal latent variables (biological, social and circumstantial) and the outcome latent variables of mental health problems and well-being. This revealed an excellent fit to the data, S-B χ^2^ (3199, N = 23,397) = 126654·8, p<·001; RCFI = ·97; RMSEA = ·04 (·038–·039). As hypothesised, a family history of mental health difficulties, social deprivation, and traumatic or abusive life-experiences all strongly predicted higher levels of anxiety and depression. However, these relationships were strongly mediated by psychological processes; specifically lack of adaptive coping, rumination and self-blame.

**Conclusion:**

These results support a significant revision of the biopsychosocial model, as psychological processes determine the causal impact of biological, social, and circumstantial risk factors on mental health. This has clear implications for policy, education and clinical practice as psychological processes such as rumination and self-blame are amenable to evidence-based psychological therapies.

## Introduction

### Mental Health and Well-being

Mental health problems affect one person in every four, making them the leading cause of disability [1] and costing an estimated $2,500 billion worldwide in 2010 [2]. The origins and phenomenology of mental disorder have provoked debate amongst researchers and practitioners for decades [3]. This is despite widespread reference to the ‘biopsychosocial model’ [4], which assumes that biological, social (environmental), circumstantial (life events), and psychological factors are all important in the aetiology of mental health problems.

It is universally accepted that biology, the environment, and adverse life events collectively cause mental problems [4]. But the precise relationship between these variables is of theoretical importance and imperative for developing effective treatment, yet continues to remain a matter of pointed scientific and professional debate [3]. One critique of the biopsychosocial model is that it fails to clarify the nature of the interrelationships between each component in the model [5]. In particular, there is little agreement over how psychological processes (e.g. behaviours, thoughts, and emotions) are implicated.

From a biological perspective, mental health problems result from genetically transmitted physical abnormalities [6], along with the additive effects of negative life-events and environmental factors, which then subsequently affect psychological functioning [7]. Genetically transmitted biological factors act via complex epigenetic interactions between genes and environmental influences from conception into adulthood which include biological (e.g. maternal stress, nutritional deficiency) as well as social (e.g. abuse, neglect, social deprivation) factors [8]. These gene-environment interactions lead to observable biochemical, structural, and functional changes in the brain [9]. However, the precise identity, nature, and function of the genes involved have yet to be identified and the effects on the brain have never been reliably demonstrated. There is also unequivocal evidence that environmental factors (e.g. poverty, unemployment, social exclusion) and a range of life events (e.g. sexual, emotional, and physical abuse) have strong associations with mental health problems [10] although, again, the precise mechanisms by which their influence accrues is not clear.

Scholarly dispute is most evident in differing accounts of the role played by psychological factors [5]. There is a wealth of evidence that core processes such as reasoning ability, thinking styles, and behaviour are important in the development and maintenance of all mental health problems [5]. Thinking styles such as self-blame and rumination are two examples of psychological processes most commonly implicated across a wide range of mental health problems [11], [12]. However, biomedical approaches suggest that biological factors have a dominant position in the cause of mental health problems and thus they are the direct result of genes or gene-environment interactions. This implies that psychological factors are symptoms or consequences of these illnesses [7], [13].

**Figure 1:**
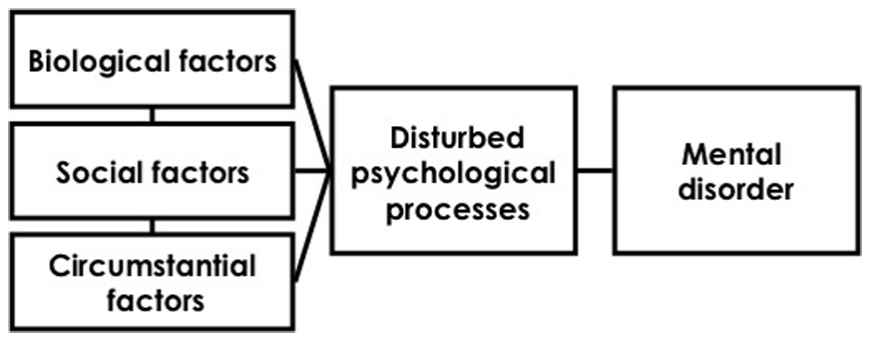
The hypothesized relationships between elements of the biopsychosocial model from Kinderman 2005. These formed the basis for our covariance modeling.

The alternative to the strictly biological view is that biological factors, social factors and other environmental or life events lead to mental health problems through their conjoint effects on psychological processes, and these are the final common pathway to mental ill-health [5] (see figure 1). This has major implications for treatment, as it would place far greater importance on evidence based psychological interventions; whereas to date, such approaches are regarded as peripheral extras to pharmacology.

Here we report on the first empirical test of the relationships between the key elements of the biospychosocial model of mental ill-health based on a representative population sample and using structural equation modeling (SEM).

## Methods

### Ethics Statement

This study complies with the guidelines of the 1964 Declaration of Helsinki. Ethical approval was obtained by the University of Liverpool’s School of Population, Community and Behavioral Science Research Ethics Committee May 2009.

#### Participants

Participants were 32,827 (age 18–85 years) self-selected respondents to an open-access online battery of questionnaires (“The Stress Test”), approved by the University of Liverpool Committee on Research Ethics and conducted in accordance with the ethical standards of the 1964 Declaration of Helsinki. In order to determine if the sample was representative of the UK population, where comparable demographic data existed, UK respondents were compared to national data [14] for England and Wales to reveal that more respondents were white, had slightly higher earnings, and were better educated than the general population, although were comparable on other demographic features. The regional breakdown was also similar to other major health surveys [15]. Demographic details are summarised in table 1.

**Table 1:** Demographics of whole sample, N = 27,397.

	*N* = 27,397
	% (*n*)
Ethnic group	
White - British, Irish, Other	92·8 (25,434)
Black Minority Ethnic	5·8 (1,612)
Rather not say or missing	1·3 (351)
Highest level of schooling achieved	
Did not complete schooling	2·2 (601)
In education until age 18	24·7 (6,766)
Degree or professional qualification	73·1 (20,030)
Occupational Status	
In education	11·4 (3,109)
In employment	73·7 (20,195)
Other	14·9 (4,093)
Total gross annual or weekly household income	
Up to £30,000 to £39,999 ($49,000–$65,000)/annum	51·9 (14,206)
Above £30,000 to £39,999 ($49,000–$65,000)/annum	36·0 (9,851)
Don’t know/prefer not to say or missing	12·1 (3,340)
Estimated parents income whilst growing up	
Lower than 50% population	50.8 (13,913)
Higher than 50% population	49·2 (13,484)
Relationship status	
In a relationship	73·2 (20,062)
Single	26·8 (7,335)
Number of children	
None	53·7 (14,717)
One or more	46·3 (12,680)

#### Procedure

The Stress Test was promoted via multi-media formats (TV, radio and online) and launched on BBC Radio 4′s ‘All in the Mind; a flagship documentary focusing on issues of the human mind. The test’s URL [www.bbc.co.uk/labuk/experiments/stress/] was publicized on radio and TV broadcasts and made available via BBC web pages and social media. The test had 12 sections, which took approximately 20 minutes to complete in total. Questionnaire items were completed in a fixed order and answers selected from a drop-down menu. Some tasks were constrained within time limits. On completion, an overview of scores was displayed on a results home-page and URL links for comprehensive and tailored feedback based on test scores were presented. Once completed, participants were not permitted to re-take the test.

#### Measures

Measures were selected on the basis of theoretical principles and empirical research to provide indicators of latent constructs representative of the components of the biopsychosocial model [4], [5]. The measurement battery was designed by authors PK and ST, and developed by all authors in collaboration with BBC Lab UK. Demographic data collected included: age; gender; ethnic group; occupation; gross annual or weekly household earnings; highest level of formal schooling; occupational status; parents’ income; relationship status; and number of children. Measured variables to represent the biological component of the theoretical model were a yes/no response to indicate participants’ reports of familial mental health diagnoses by a psychiatrist or GP [16], and performance on two cognitive tests to detect response to negative feedback and negative and positive stimuli. These were the ‘delayed match to sample’ and the ‘affective go no go’ tasks adapted from the Cambridge Neuropsychological Test Automated Battery [17], [18], [19]. The social inclusion component of the model was represented by an 11-item questionnaire indicating social relationships with friends and family, and participation in social activities [20]. These were a combination of Likert scale and yes/no responses.

Indicators of the circumstantial component included recent life events measured using the List of Threatening Experiences Questionnaire [21]. and historical life events measured using a 5-point Likert scale of which participants indicated if they believed they had historically been physically, sexually, or emotionally abused, or bullied at school [22]. The first of the two key psychological processes, response style, was measured using an adapted Response Style Questionnaire [12], where participants indicated on a Likert scale their response to stressful situations from a list of coping strategies pertaining to rumination, problem solving/adaptive, or dangerous activities. The second, attributional style, was measured using a modified version of the Internal, Personal and Situational Attributions Questionnaire [23] to determine the degree to which individuals generate internal, personal, or situational causes for hypothetical negative events. Finally, mental health problems were assessed by the Goldberg Anxiety and Depression Scales [24] and the BBC Well-being Scale [25].

## Results

### Data Analysis

SEM relies on the identification and subsequent analysis of latent variables or factors [26], which represent underlying theoretical constructs that cannot directly be measured, to explore and test the simultaneous patterns of causal influence and response among multiple variables [27].

A two-step analytical approach was used, conducted using the EQS structural equation modeling (SEM) program [28]. First, missing data were deleted listwise, yielding complete data on 19,966 participants (retention of 60·8% of the original sample). However, the neurocognitive data accounted for a large proportion of missing data due to the invalid recording of data, likely due to technical error, With the exclusion of the neurocognitive data, listwise deletion of the remaining variables provided a sample of 27,397 (retention of 83% of the original sample). Analysis revealed no significant differences between those with and without missing data on demographic variables and a selection of measured variables.

Because of the multivariate kurtosis in the data, goodness of fit of models was evaluated with the adjusted robust comparative fit index (RCFI) based on the Satorra-Bentler χ^2^ statistic [29]. There is no absolute consensus on these matters, so, in accordance with more conservative recommendations [26], we used a ratio of χ^2^ to degrees of freedom of less than 2·0, a comparative fit index of greater than ·90 [30], and a Root Mean Square Error of Approximation (RMSEA) of less than ·05 [30]. As the χ^2^statistic is dependent on sample size, and likely to reject well-fitting models in large samples such as ours, we therefore concentrated on RCFI and the RMSEA indices to establish the validity of the model. An initial confirmatory factor analysis (CFA) was performed to assess the adequacy of the proposed factor structure and the relationships between latent and measured variables [31]. Where the hypothesised factor structure yielded an inadequate fit of the data, model modifications were made consistent with theoretical and conceptual assumptions of the measured variables [32].

In the development of the model path, elimination was monitored via successive improvement of the χ^2^, RCFI, and RMSEA statistics. This ‘measurement model’ phase of analysis will be reported in detail elsewhere (“Establishing the construct validity and factor structure of latent psychosocial variables in psychiatric research”, Pontin et al., submitted). Once the factor structure was established, the predictive path model was tested whereby the latent variables of psychological processes were positioned as a mediating variable between the causal latent variables (biological, social and circumstantial) and the outcome latent variables of mental health problems and well-being.

### Direct and Mediated Paths to Well-being and Mental Health Problems

**Figure 2:**
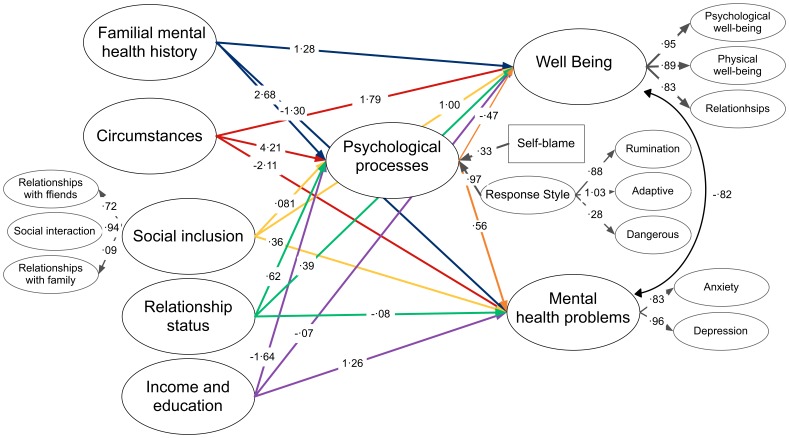
Psychological processes mediate the impact of familial risk, social circumstances and life events on mental health. Results of a structural equation model testing the mediating effects of the psychological processes of response style and self-blame on the contribution of familial mental health history, relationship status, income and education, social inclusion and life events on mental health problems and well-being, with S-B χ^2^ (3,199, N = 27,397) = 126,654·8, p<·001; RCFI = ·97; RMSEA = ·04 (·038–·039). The path diagram shows completely standardised robust parameter estimates which represent the relative contribution of each latent factor to the model. All coefficients are statistically significant, p<·001. Latent factors are represented by ovals. The double headed arrow between mental health problems and well-being represents the correlations between these latent constructs.

The initial CFA established that we had a robust measurement model with latent factors comprising all of the key components of the hypothesis under test, S-B χ^2^ (3,199, N = 27,397) = 126,654·8, *p*<·001; RCFI = ·97; RMSEA = ·04 (·038–·039). These latent factors are listed in tables 2, 3, 4 and 5 together with their standardised factor loadings, and can also be seen in figure 2.

**Table 2:** Measured variables and latent factors (causal factors).

Latent factors & measured variables	Standardised loading
*Biological*	
**Familial Mental Health Diagnosis**	
Mother diagnosed with a mental health problem	·46
Father diagnosed with a mental health problem	·33
Sibling diagnosed with a mental health problem	·48
More than sibling diagnosed with a mental health problem	·38
*Social*	
**Relationships with Friends**	
Relationship with friends	·90
See other relative/friend weekly	·43
**Relationships with Family**	
Relationship with family	4·77
See parent weekly	·06
See sibling weekly	·05
**Social Interactions**	
How do you best describe your social activities	·87
Attend an evening class	·12
Given up time for charity or local group	·27
Involved in club/organisation/religious group	.33
Participated in sports/physical activity	·37
Go to the cinema	·28
*Circumstantial*	
**Life Circumstance**	
In the past I believe I was physically abused	·49
In the past I believe I was sexually abused	·35
In the past I believe I was emotionally abused	·67
In the past I believe I was bullied at school	·44
Total number of life-events	·55
*Demographic*	
**Income/Education**	
Parental income	·37
Current income	·30
Educational attainment	·37
**Relationship status and children**	
Relationship status	·61
Number of children	·41

**Table 3:** Measured variables and latent factors (mediating psychological factors).

Latent factors & measured variables	Standardised loading
*Psychological Processes - Response Style*	
**Rumination**	
Think of shortcomings, failings, faults & mistakes	·68
Think about how angry with self	·65
Think about something to make myself feel better	·29
Think about how passive & unmotivated you feel	·75
Try to understand self by focusing on depressed feelings	·61
Isolate yourself and think of reasons feel sad	·63
Think about how you don’t feel up to doing things any more	.80
**Adaptive/Problem Solving**	
Do something that has made feel better in past	·64
Think I’m going to do something to make myself feel better	·60
Make a plan to overcome a problem	·58
Try to understand self by focusing on depressed feelings	·30
Remind yourself that feelings won’t last	·51
**Dangerous Activities**	
Drink alcohol excessively	·47
Take recreational drugs	·32
Do something reckless or dangerous	·56
*Psychological Processes – Attributional Style*	
Internal attributions (self-blame)	.33

**Table 4:** Measured variables and latent factors (mental health problems).

Latent factors & measured variables	Standardised loading
*Mental Disorder – Anxiety & Depression*	
**Anxiety**	
Have you felt anxious or on edge	·57
Have you been worrying a lot	·67
Have you been irritable	·53
Have you had difficulty relaxing	·66
Have you been sleeping poorly	·46
Have you had a headache or neck ache	·35
Trembling/tingling/dizzy spells/sweating/	·47
Have you been worried about your health	·48
Have you had difficulty falling asleep	·41
**Depression**	
Have you had low energy	·56
Have you had loss of interest	·64
Have you lost confidence in yourself	·70
Have you felt hopeless	·70
Have you had difficulty concentrating	·58
Have you lost weight (due to poor appetite)	·22
Have you been waking early	·19
Have you felt slowed up	·58
Have you tended to feel worse in the morning	·38

**Table 5:** Measured variables and latent factors (well-being).

Latent factors & measured variables	Standardised loading
*Well-being*	
**Psychological Well-being**	
Do you feel depressed or anxious ?	·04
Do you feel able to enjoy life	·80
Do you feel you have a purpose in life	·70
Do you feel optimistic about the future	·76
Do you feel in control of your life	·78
Do you feel happy with yourself as a person	·80
Are you happy with your looks and appearance	·60
Do you feel able to live your life the way you want	·77
Are you confident in your own opinions and beliefs	·54
Do you feel able to do the things you choose to do	·71
Do you feel able to grow and develop as a person	·73
Are you happy with yourself and achievements	·72
Are you happy with friendships/relationships	.16
**Physical Health and Well-being**	
Are you happy with your physical health	·63
Are you happy with the quality of your sleep	·60
Are you happy with your ability to perform daily living activities	·77
Are you happy that you have enough money to meet your needs	·51
Are you happy with your opportunity for exercise/leisure	·59
Are you happy with access to health services	·48
Are you happy with your ability to work	·65
**Relationships**	
Are you happy with your personal and family life	·74
Are you happy with your friendships and personal relationships	·93
Are you comfortable about way you relate connect with others	·74
Are you happy with your sex life	·50
Are you able to ask someone for help with a problem	·67

Notes: Standardised loadings of measured variables on their respective latent factors for the structural model, S-B χ^2^ (3,199, N = 27,397) = 126,654·8, *p*<·001; RCFI = ·97; RMSEA = ·04 (·038–·039). Components of the biopsychosocial model are shown in italics; latent factors in bold. All coefficients are statistically significant, p<·0001.

### Structural Model: Direct and Mediated Paths to Well-being and Mental Health Problems

The second step in the analysis tested how the latent factors revealed in the CFA to represent key elements of the biopsychosocial model [4] were related to mental health problems and well-being, and to test the hypothesised mediating role of psychological processes [5].

Initially, we tested a default model, exploring the relationships between putative causal factors (familial mental health history, relationship status, income and education, social inclusion and life events) with well-being and mental health problems, without the mediating role of psychological processes. This revealed a poor fit to the data, χ^2^ (3,205, *N* = 27,397) = 168355·3, *p*<·001; RCFI = ·78; RMSEA = ·04 (·043–044).

Next, we used SEM to test a model with the same latent factor predictors, but including the hypothesised mediating role of psychological processes (see figure 2), and conducted on the 23,397 participants with complete datasets for these variables. This revealed an excellent fit to the data, anxiety, S-B χ^2^ (3199, N = 23,397) = 126654·8, *p*<·001; RCFI = ·97; RMSEA = ·04 (·038–·039). All parameter estimates are shown in figure 2.

Structural equation models can be used to infer causality more robustly than conventional correlational analyses, as they account for interactions between factors [27]. In our results, there was a significantly improved model fit following the insertion of the psychological processes factor as a mediator of the relationship between the known causal factors and mental health and well being. Exploration of the direct and mediated paths also strongly supports the significant mediating role of psychological processes in the causation of mental health problems (see table 6) and poorer well-being (see table 7), illustrated by the strength of the mediator expressed in the path parameters.

**Table 6:** Direct and mediated predictors of mental health problems.

	Direct	Mediated	Total
Familial mental health history	1·30	1·50	2·80
Relationship status	0·08	0·39	0·43
Income and education	1·26	0·92	2·18
Social inclusion	0·36	0·04	0·40
Life events	2·11	2·36	4·47

Notes: Parameter estimates representing the effects of familial mental health history, relationship status, income and education, social inclusion and life events on mental health problems, with and without the mediating effect of psychological processes of response style and self-blame.

**Table 7:** Direct and mediated predictors of well-being.

	Direct	Mediated	Total
Familial mental health history	1·28	1·26	2·54
Relationship status	0·39	0·29	0·68
Income and education	0·07	0·77	0·84
Social inclusion	1·00	0·04	1·04
Life events	1·79	1·98	3·77

Notes: Parameter estimates representing the effects of familial mental health history, relationship status, income and education, social inclusion and life events on well-being, with and without the mediating effect of psychological processes of response style and self-blame.

These results show that life events (childhood abuse and bullying, and stressful life events in adulthood) were the strongest direct predictors of mental health problems (depression and anxiety). A familial history of mental health problems and social status (income and education) were the next most significant direct predictors of mental health problems - and here it should be remembered that genetic or biological factors are not the only vectors for the familial transmission of mental health problems [3]. Social inclusion and relationship status were also significant direct predictors of mental health problems.

As hypothesised, however, the key psychological processes of response style and self-blame were significant mediators of all these paths. The overall fit of the model - its ability to explain the data reported in this population – was significantly improved by the inclusion of psychological processes as mediators in the hypothesised relationship between biological factors, life events, and environmental challenges, and mental health and well being. Moreover, life events and familial mental health history were the most significant direct predictors of mental health problems. However, the causal pathways involving the mediation of response style and self-blame were stronger predictors than direct paths. This was also true for the (smaller) effect of relationship status. The direct effects of social status (income and education) and social inclusion on mental health problems remained more significant than the mediated routes, but in each case there was a significant mediation effect.

A broadly similar pattern was observed in the prediction of well-being. Again, life events were the strongest predictors of well-being, followed by a familial history of mental health problems and social inclusion. Again, psychological processes were very significant mediating factors. This mediation effect was most significant in the path involving life-circumstances and social status (income and education).

## Discussion

### Social Determinants of Mental Health Problems, and Psychological Therapies

Our results demonstrate that psychological processes of response style (specifically a greater tendency to ruminate) and self-blame (or an internal attributional style for negative events) powerfully determine the impact of familial histories of mental health problems, life events and traumas, and social deprivation in the aetiology of depression and anxiety and in the maintenance of well-being. This study is the first multivariate empirical test of specific and previously published hypotheses [5] about the role of psychological processes as mediators in a revision of the ubiquitous bio-psycho-social model [4]. Our access to this unprecedented and large data set has allowed clear dissection of the inter-connections between factors, and in particular, has permitted analysis of the specific mediating effect of psychological factors. Our results clearly support the contention that biological, social, and circumstantial causal agents affect our mental health and well-being through their impact on how we process information and perceive the world. In this study, life events constituted the most significant direct causal factor, and two key processes – self-blame and response style – significantly mediated all causal pathways.

Our results did not support a fully mediated model (that is, with no residual direct effects), but this is entirely unsurprising. Mental health and well-being can be safely assumed to be the result of a huge number of causal factors with a large number of mediating psychological processes. In this study, we examined only two of the very many psychological processes hypothesised to be important in mental health. Nevertheless, we are confident both that these findings are themselves robust and that other psychological processes would also act as mediators in causal paths similar to those revealed here.

The present study was designed as an empirical test of a hypothesised set of relationships derived from previously published theoretical research [5]. Because of this, and for practical reasons, we reduced the huge complexity of mental health to a testable model of linear relationships between a limited number of variables. Mental health problems, like all other clinical conditions, can be understood on many simultaneous levels, incorporating genetic, metabolic, cellular, systemic bodily, personal, social anthropological and spiritual dimensions. We did not, for example, address issues concerning individuals’ understanding of their own mental health issues [33], nor did we dissect the complex relationships between genetics, heritability and family history [34]. Further research is clearly required to explore how the detailed pathway from genetics through neurocognitive processes on the one hand, and interpersonal and interpretative frameworks on the other, link to mental health outcomes. Anxiety and depression are recognised as two major dimensions underlying common mental health problems, but there are clearly very many more recognised psychological difficulties. Further research could also explore whether different psychological mechanisms mediate the pathways from either specific or generic causal and risk factors to different mental health problems.

There was also a potential element of self-selection in the present study, given the recruitment strategy and the on-line methodology. However, although more of our participants were white, had slightly higher earnings, and were better educated than the England and Wales average [14], there was broad comparability with other national demographic data, with a similar regional breakdown to other major health surveys [15].

These results support a significant revision of the biopsychosocial model. Instead of regarding these three causal agents as co-equal partners in the aetiology of mental health problems, these results demonstrate that the impact of physical and social causes on mental health and well-being outcomes is mediated by psychological processes. In other words, psychological processes determine the causal impact of biological, social, and circumstantial risk factors.

These findings and this interpretation have significant implications. Reductionist biological accounts of mental health have been robustly criticised on scientific, ethical, and practical grounds [3]. An alternative, scientifically valid, model may have implications for policy, education and clinical practice [5], [33]. Psychological processes such as rumination and self-blame are amenable to evidence-based psychological therapy [35]. Significant gains in mental health are achieved when people experiencing mental health problems are supported in achieving greater control over their own psychological processes [36]. A clear understanding of the role of psychological processes in the aetiology of mental health problems and the maintenance of well-being is an important step in that process. Further research in this area should include further validation of this model (particularly through prospective studies), careful consideration of the interactions between causal factors (particularly biological factors) and the mediating role of psychological mechanisms.
